# Biallelic *RFX6* mutations can cause childhood as well as neonatal onset diabetes mellitus

**DOI:** 10.1038/ejhg.2015.161

**Published:** 2015-08-12

**Authors:** Francis H Sansbury, Birgül Kirel, Richard Caswell, Hana Lango Allen, Sarah E Flanagan, Andrew T Hattersley, Sian Ellard, Charles J Shaw-Smith

**Affiliations:** 1 Institute of Biomedical and Clinical Science, University of Exeter Medical School, Exeter, UK; 2 Endocrinology Unit, Faculty of Medicine, Department of Pediatrics, Eskişehir Osmangazi University, Eskişehir, Turkey

## Abstract

Neonatal diabetes is a highly genetically heterogeneous disorder. There are over 20 distinct syndromic and non-syndromic forms, including dominant, recessive and X-linked subtypes. Biallelic truncating or mis-sense mutations in the DNA-binding domain of the RFX6 transcription factor cause an autosomal recessive, syndromic form of neonatal diabetes previously described as Mitchell–Riley syndrome. In all, eight cases have been reported, with the age at onset of diabetes in the first 2 weeks of life. Here we report two individuals born to double first cousins in whom intestinal atresias consistent with a diagnosis of Mitchell–Riley syndrome were diagnosed at birth, but in whom diabetes did not present until the ages of 3 and 6 years. Novel compound heterozygous *RFX6* nonsense mutations (p.Arg726X/p.Arg866X) were identified at the 3′ end of the gene. The later onset of diabetes in these patients may be due to incomplete inactivation of RFX6. Genetic testing for *RFX6* mutations should be considered in patients presenting with intestinal atresias in the absence of neonatal diabetes.

## Introduction

Neonatal diabetes is defined as diabetes diagnosed within the first 6 months of life. It is highly genetically heterogeneous, with dominant, recessive and X-linked patterns of inheritance well described, comprising to date over 20 distinct syndromic and non-syndromic forms with a combined incidence of around 1 in 100 000 births.^1^

During early childhood, type 1 diabetes predominates. However, monogenic causes of diabetes should also be considered in this group. Examples of syndromic monogenic diabetes in this age range include Wolfram syndrome,^2^ thiamine-responsive megaloblastic anemia syndrome^3^ and diabetes due to heterozygous mutations in the transcription factor GATA6, a cause of neonatal diabetes in association with congenital heart disease and other malformations.^4^

Biallelic mutations in *RFX6* (regulatory factor X, 6) cause neonatal diabetes mellitus in association with intestinal atresias, and hepatobiliary abnormalities. This condition has been designated as Mitchell–Riley syndrome.^5^ To date there have been eight genetically confirmed cases, comprising seven probands and one sibling.^5, 6, 7, 8, 9, 10^ Typically, fetal abnormality is suspected pre-natally and a diagnosis of duodenal and/or jejunal atresia confirmed shortly after birth. Low birth weight and ongoing feeding difficulties relating to bowel surgery contribute to failure to thrive. Cholestasis, partly secondary to total parenteral nutrition and partly due to a poorly defined primary intrahepatic cholangiopathy may result in hepatic failure. Diabetes is diagnosed within the first few weeks of life. Prognosis is poor, with death occurring within the first 6 months in five of the cases described to date.

Here, we report the first cases of Mitchell–Riley syndrome in which the onset of diabetes occurred outside the neonatal period. We compare their clinical and molecular characteristics with the eight previously reported cases.

## Methods

### Genetic testing

Genomic DNA was extracted by standard protocols and the proband was tested for mutations in all known neonatal diabetes genes using a previously described targeted next generation sequencing assay.^11^ Targeted sequence capture utilized a custom cRNA bait library (Agilent, Santa Clara, CA, USA) to enrich for exons and flanking sequences from 29 genes, including *RFX6*, in which mutations are known to cause monogenic forms of diabetes.^11^ Potential mutations were confirmed using Sanger sequencing.

### Literature review

Cases of Mitchell–Riley syndrome were identified from Pubmed (http://www.ncbi.nlm.nih.gov/pubmed). Those cases in whom confirmatory biallelic mutations in *RFX6* had been identified were included in our review.

## Clinical reports and results

**Figure 1:**
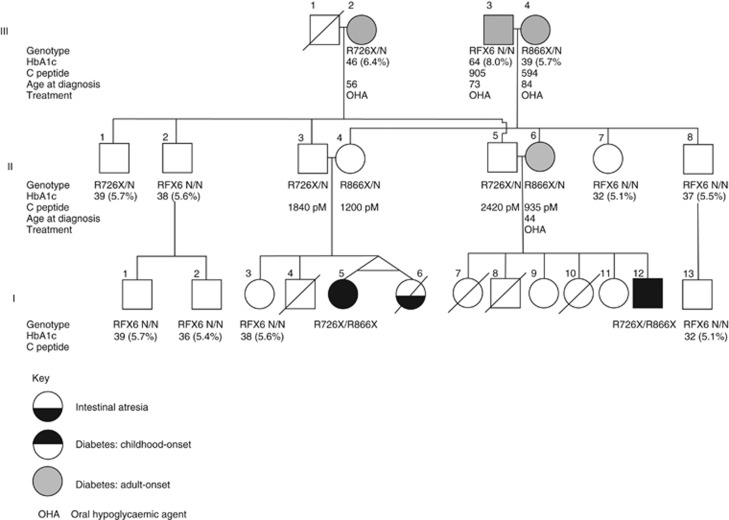
Pedigree. Refer to key for interpretation of symbols. *RFX6* genotypes are shown underneath relevant family members. HbA1c (units: mmols/mol(%)) and C-peptide values (pM) are given for selected family members. See text for further explanation.

Case 1 (Figure 1, I5), current age 9 years, is a female born at 32 weeks' gestation, birth weight 1.65 kg (42nd centile), with duodenal atresia, jejunal web and Meckel's diverticulum. She underwent operative intervention for these malformations before being discharged from hospital at 3 months of age. During an admission at the age of 2 years, hyperglycemia (10.6 mmol/l, random sample) had been noted. However, no further investigation or treatment was put in place at that time. She presented at the age of 3 years with diabetic ketoacidosis. Physical examination revealed no abnormalities other than those associated with elevation of blood glucose at 33.0 mmol/l. Her body weight was 18.5 kg (10–25th centile). Height was 118 cm (25–50th centile). Investigations showed insulin <2 μI U/ml, C-peptide 0.023 ng/ml, blood pH: 7.40; urine ketone: +++, HbA1c 8.96%, anti-GAD, IAA, ICA negative. She was prescribed a dose of 0.35 U/kg per day of long-acting insulin with occasional bolus insulin.

Otherwise, her past medical history was notable for multiple hospital admissions due to gastrointestinal bleeding. At the age of 5 years, she underwent a small intestinal resection, histological examination of which revealed the presence of patches of gastric heterotopia within the small bowel. The gastro-intestinal bleeding subsequently resolved.

Additional investigation revealed absent gall bladder with otherwise normal biliary tract. Liver function tests have always been within normal limits.

She has a healthy 16-year-old sister. A brother died of prematurity and meconium aspiration at 1 week of age. An identical twin was diagnosed with duodenal atresia and underwent corrective surgery at 2 days of age. She died of complications of prematurity and surgery at the age of 1 month.

Case 2 (Figure 1, I12), her double first cousin, current age 9 years, is a male infant who was born at 34 weeks gestation, birth weight 1.70 kg (7th centile), with duodenal atresia and mid-gut malrotation. He was diagnosed with diabetes having presented with hyperglycemia (16.0 mmol/l) at the age of 6 years. He had no prior symptoms suggestive of diabetes and hyperglycemia had been identified at home by his mother. He did have a history of asymptomatic fasting hyperglycemia (7.2 mmol/l) determined 1 year previously in another hospital; no treatment was initiated at that time.

Physical examination was unremarkable. His body weight was 17.5 kg (10th centile). Height was 113 cm (25th centile). Investigations at this time showed: blood glucose 16.0 mmol/l; insulin: <2 μI U/ml; C-peptide 0.39 ng/ml, blood: pH: 7.41; urine ketones: negative; and HbA1C: 7.96%. Anti-GAD, IAA and ICA antibodies negative. His current insulin requirement is ~0.7 units/kg per day (3 × 3–4 unit humalog before meals and 4 units insulin glargine before bed. His last recorded HbA1c level was 7.2%.

His past history is notable for chronic iron deficiency anemia. Despite extensive investigation at two different hospitals, it has not been possible to identify a cause for this. No structural abnormality of the hepato-biliary tract has been identified, and his liver function tests have always been within normal limits.

He has healthy sisters, current ages 20 and 11 years. Another sister died of complications of cerebral palsy at the age of 17 years. A brother died of complications of prematurity at 1 week of age. His mother was diagnosed with diabetes 4 years previously.

There was no history of parental consanguinity. However, the mothers of the two cases are sisters and the fathers are brothers, making the affected individuals double first cousins.

**Figure 2:**
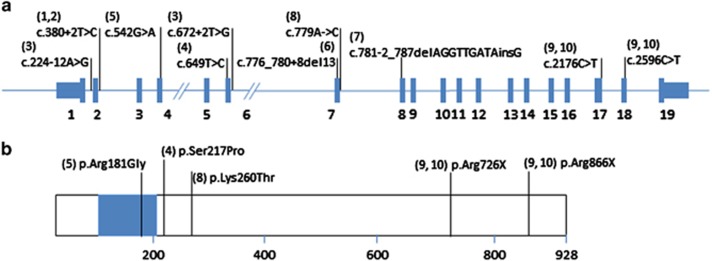
(**a**) *RFX6* gene structure. Exons are shown in blue with exon numbers underneath. Mutations are shown preceded by a number in brackets which denotes the case in Table 1 to which it refers. (**b**) RFX6 protein schematic. The DNA-binding domain is shown as a blue box. Amino acid numbering is shown, together with aminoc acid changes due to mis-sense or nonsense mutations. The number in parenthesis preceding each amino acid change denotes the case in Table 1 to which it refers. The mutations in the patients who are the subjects of the present report are the only ones so far reported in any of exons 9–19, all others occurring in exons 1–8, or the intervening introns. A full color version of this figure is available online at the *European Journal of Human Genetics* website.

Genetic testing showed that both children are compound heterozygotes for the *RFX6* nonsense mutations c.2176C>T (p.Arg726X) and c.2596C>T (p.Arg866X) in exons 17 and 18, respectively (Figure 2). The fathers were heterozygous for p.Arg726X and the mothers heterozygous for p.Arg866X. Further testing demonstrated that the mutations had been inherited from both grandmothers.

Other family members had been diagnosed with diabetes in adulthood, as shown in the pedigree and in Table 1. The mother of case 2, both grandmothers and the surviving (maternal) grandfather (Figure 1, II6, III2, III4 and III3, respectively) developed adult-onset diabetes. Age of diagnosis for these four individuals is provided in Figure 1. All four are non-obese (BMI data not available) and treated with an oral hypoglycaemic agent. C-peptide and HbA1c levels were assayed in all four heterozygote parents and in both heterozygote grandmothers, and, for comparison, in one further non-diabetic heterozygote and a further eight non-diabetic family members without a mutation (see Figure 1). For heterozygotes in the family, there was no significant difference in C-peptide levels between those who had and had not developed diabetes. There was also no difference in HbA1c between the non-diabetic heterozygotes and those who are negative for the relevant familial mutation. Taking these data together, there is no support for the idea that being heterozygous for either *RFX6* mutation is a risk factor for abnormal HbA1c or C-peptide levels, or diabetes. It remains possible that heterozygous carriers of RFX6 5′ mutations may have increased risk of early-onset type 2 diabetes. This could be evaluated in a larger cohort by testing of beta cell function as a function of age in these subjects in comparison with controls.

A review of the clinical phenotype observed in previously published cases is shown in Table 1. All cases with a molecularly confirmed diagnosis of Mitchell–Riley syndrome are included, together with the present cases. The eight previously published cases demonstrate a high degree of phenotypic concordance. Duodenal atresia is a present in all cases, with or without jejunal atresia/mid-gut malrotation. Gall-bladder agenesis is present in 7/8. All cases had evidence of liver involvement with intrahepatic cholestasis in 7/8 cases and ascites in a further case. Five of the eight children had died, all within the first year of life. Aside from the difference in age of onset of diabetes in the two new cases, the phenotype differed from previously published cases in the absence of any liver pathology, although one of the cases (case 1) had absent gall bladder. This relatively milder phenotype is reflected in the greater age of survival of the two cases in the present report.

## Discussion

We report double first cousins in whom the identification of biallelic *RFX6* mutations has confirmed a diagnosis of Mitchell–Riley syndrome. These new cases are the first with Mitchell–Riley syndrome in whom the diagnosis of diabetes was not made in the neonatal period, all other cases to date having been diagnosed within the first 2 weeks of life. The diagnosis of diabetes in these cousins at the ages of 3 and 6 years is therefore significantly different from the previously expected phenotype. In both of the cousins, a period of asymptomatic hyperglycemia of at least 1 year preceded a formal diagnosis of diabetes.

The results of mutation analysis of the eight molecularly confirmed probands (excluding those in the present report) suggest a possible genotype/phenotype correlation. Among the nine mutations identified in these eight cases (one a compound heterozygote, the remaining seven homozygous), six affect splicing and/or the reading frame in exons 2–8; the remaining three are mis-sense mutations clustering within or close to the DNA-binding domain (Figure 2). The cases in the present report are compound heterozygotes for premature truncating mutations in exons 17 and 18 of the 19 exon *RFX6* gene, the first such mutations in *RFX6* to be described. The exon 18 mutation, c.2596C>T (R866X) is predicted to escape nonsense-mediated decay because it lies within 50 nucleotides of the last exon–exon boundary of the gene. A partially truncated version of the wild-type protein, which comprises 928 amino acids, is therefore predicted to be produced, possibly with some function given the full preservation of the DNA-binding domain. The second mutation, c.2176C>T (R726X), is predicted to be subject to nonsense-mediated decay.

There are reports in the literature of distinct phenotypes associated with mutated transcripts depending upon the presence or absence of nonsense-mediated decay. 3′ mutations in *SOX10* result in a severe neurocristopathy designated PCWH.^12^ More 5′ mutations result in the more restricted phenotype of Waardenburg–Shah syndrome. Transcripts with 3′ mutations escape nonsense-mediated decay and in this instance the phenotype is postulated to be more severe than that due to more 5′ mutations because failure of the NMD pathway results in production of a protein with a dominant negative effect. A distinct clinical phenotype is also postulated in instances of 3′ mutations in the *FBN1* gene, with the same proposed mechanism of escape from NMD.^13^

In the present case of biallelic mutations in *RFX6*, the transcript with the 3′-most *RFX6* mutation (c.2596C>T) is predicted to escape NMD, a possibility which could be tested by analyzing levels of wild-type and mutated transcripts in a suitable experimental assay. It is possible that escape from NMD could lead to some retention of function of the RFX6 protein generated from that allele, with a resulting milder phenotype with respect to age of onset of diabetes and other clinical features.^14^

The data presented support the idea that biallelic mutations in *RFX6* should be considered in any infant presenting with duodenal or jejunal atresia with or without intestinal malrotation, especially in the context of possible autosomal recessive inheritance. A genetic diagnosis confers not only the advantage of establishing a molecular genetic diagnosis, but also alerts the clinician to the future possibilities of onset of diabetes during infancy or childhood, as well as possible hepatobiliary complications.

In summary, we report two individuals each with compound heterozygous *RFX6* nonsense mutations and an intestinal phenotype consistent with Mitchell–Riley syndrome, but with onset of diabetes in childhood rather than in the first 2 weeks of life. Genetic testing for *RFX6* mutations should be considered in patients presenting with intestinal atresias in the absence of neonatal diabetes, especially where sibling recurrence or parental consanguinity point to possible autosomal recessive inheritance.

## Figures and Tables

**Table 1:** Details of individuals with biallelic mutations in *RFX6*

*Case no.*	*Relationship*	*Proband no. in Smith* ^(ref. 5)^	*Sex*	*Ethnic origin*	*Birth weight*	*Gestational age at birth*	*Age of onset of diabetes*	*GI atresia/malrotation*	*Hepato- biliary*	*Pancreas*	*Other*	*Outcome*	*RFX6 mutation- nucleotide*	*RFX6 mutation- protein*	*Reference*
1	n/a	1	M	Pakistani	1540 g	36 weeks	Day 1	DA, JA	GBA	AP	Malabsorption unresponsive to pancreatic supplements/bile acids; cholestasis	Deceased at 158 days	c.380+2T>C homozygous	p.?	Mitchell *et al.*^6^
2	Sibling of case 1	n/a	F	Pakistani	1310 g	34 weeks	Day 2	DA, JA	GBA	AP	Duodenal biopsy: partial villous atrophy. Intrahepatic cholestasis.	Deceased at 194 days	c.380+2T>C homozygous	p.?	Mitchell *et al.*^6^
3	n/a	3	F	Not stated	2295 g	39 weeks	Day 2	Duodenal web and malrotation	GBA	Small Pancreas	Intrahepatic cholestasis; bilateral inguinal hernias	Alive at 1 year; HbA1c 7.5% off insulin treatment	c.672+2T>G/c.224-12A>G compound heterozygote	p.?/p.?	Mitchell *et al.*^6^
4	n/a	2	F	Pakistani	1700 g	35 weeks	Day 8	DA, malrotation	GBA	Undetectable fecal elastase	Intrahepatic cholestasis; anteriorly placed anus	Alive at 1 year 9 months	c.649T>C homozygous	p.Ser217Pro	Chappell *et al.*^7^
5	n/a	5	M	Not stated	1340 g	38 weeks	Soon after birth	DA, JA (apple peel-type), intestinal malrotation	GBA	Pancreatic hypoplasia	Intrahepatic cholestasis; malabsorption unresponsive to pancreatic supplements/bile acids; neonatal haemochromatosis	Deceased at 2 months	c.542G>A homozygous	pArg181Gly	Martinovici *et al.*^8^
6	n/a	4	M	French, non-consanguineous	<10th centile	35 weeks	Day 2	DA	No abnormality reported	No abnormality reported	Ascites, sepsis, gastro-intestinal haemorrhage	Deceased at 2.5 months	c.776_780+8del13 homozygous	p.?	Smith *et al.*^5^
7	n/a	n/a	M	Arab-Israeli	1490 g	38 weeks	Day 1	DA, JA, intestinal malrotation	GBA	AP	Intrahepatic cholestasis; red cell aplasia confirmed on bone marrow biopsy; malabsoprtion unresponsive to pancreatic supplements/bile acids	Alive at 1 year 9 months, on twice daily insulin, HbA1c 7.1%	c.781-2_787delAGGTTGATAinsG homozygous	p.?	Spiegel *et al.*^9^
8	n/a	n/a	M	Vietnamese	1375 g	34 weeks	Day 1	DA, intestinal malrotation	GBA	AP	Intrahepatic cholestasis; malabsoprtion unresponsive to pancreatic supplements/bile acids	Deceased at 5 months	c.779A>C homozygous	p.Lys260Thr	Concepcion *et al.*^10^
9	n/a	n/a	F	Turkish	1650 g	32 weeks	3 years	DA, jejunal web, Meckel's diverticulum	GBA	No abnormality reported	No abnormality reported	Alive at 9 years	c.2176C>T homozygous	p.Arg726X	Present report
10	Double-first cousin of case 9	n/a	M	Turkish	1700 g	34 weeks	6 years	DA, mid-gut malrotation	No abnormality reported	No abnormality reported	No abnormality reported	Alive at 9 years	c.2176C>T homozygous	p.Arg726X	Present report

Abbreviations: GBA, gall bladder agenesis; DA, duodenal atresia; JA, jejunal atresia; AP, annular pancreas.

## References

[bib1] Iafusco D, Massa O, Pasquino B et al: Minimal incidence of neonatal/infancy onset diabetes in Italy is 1:90,000 live births. Acta Diabetol 2012; 49: 405–408. 21953423 10.1007/s00592-011-0331-8 PMC3464369

[bib2] Barrett TG, Bundey SE: Wolfram (DIDMOAD) syndrome. J Med Genet 1997; 34: 838–841. 9350817 10.1136/jmg.34.10.838 PMC1051091

[bib3] Shaw-Smith C, Flanagan SE, Patch AM et al: Recessive SLC19A2 mutations are a cause of neonatal diabetes mellitus in thiamine-responsive megaloblastic anaemia. Pediatr Diabetes 2012; 13: 314–321. 22369132 10.1111/j.1399-5448.2012.00855.x

[bib4] Lango Allen H, Flanagan SE, Shaw-Smith C et al: GATA6 haploinsufficiency causes pancreatic agenesis in humans. Nat Genet 2011; 44: 20–22. 22158542 10.1038/ng.1035 PMC4062962

[bib5] Smith SB, Qu HQ, Taleb N et al: Rfx6 directs islet formation and insulin production in mice and humans. Nature 2010; 463: 776. 10.1038/nature08748 PMC2896718 20148032

[bib6] Mitchell J, Punthakee Z, Lo B et al: Neonatal diabetes, with hypoplastic pancreas, intestinal atresia and gall bladder hypoplasia: search for the aetiology of a new autosomal recessive syndrome. Diabetologia 2004; 47: 2160. 15592663 10.1007/s00125-004-1576-3

[bib7] Chappell L, Gorman S, Campbell C et al: A Further Example of a Distinctive Autosomal Recessive Syndrome Comprising Neonatal Diabetes Mellitus, Intestinal Atresias and Gall Bladder Agenesis. Am J Med Genet 2008; 146A: 1713. 18512226 10.1002/ajmg.a.32304

[bib8] Martinovici D, Ransy V, Vanden et al: Neonatal hemochromatosis and Martinez-Frias syndrome of intestinal atresia and diabetes mellitus in a consanguineous newborn. Eur J Med Genet 2010; 53: 25. 19887127 10.1016/j.ejmg.2009.10.004

[bib9] Spiegel R, Dobbie A, Hartman C et al: Clinical characterization of a newly described neonatal diabetes syndrome caused by RFX6 mutations. Am J Med Genet 2011; 155A: 2821. 21965172 10.1002/ajmg.a.34251

[bib10] Concepcion JP Reh CS, Daniels M et al: Neonatal diabetes, gallbladder agenesis, duodenal atresia, and intestinal malrotation caused by a novel homozygous mutation in RFX6. Pediatr Diabetes. 2014; 15: 67–72. 23914949 10.1111/pedi.12063 PMC3871990

[bib11] Ellard S, Lango Allen H, De Franco E et al: Improved genetic testing for monogenic diabetes using targeted next-generation sequencing. Diabetologia 2013; 56: 1958–1963. 23771172 10.1007/s00125-013-2962-5 PMC3737433

[bib12] Inoue K, Khajavi M, Ohyama T et al:: Molecular mechanism for distinct neurological phenotypes conveyed by allelic truncating mutations. Nat Genet 2004; 36: 361–369. 15004559 10.1038/ng1322

[bib13] Horn D, Robinson PN: Progeroid facial features and lipodystrophy associated with a novel splice site mutation in the final intron of the FBN1 gene. Am J Med Genet A 2011; 155A: 721–724. 21594993 10.1002/ajmg.a.33905

[bib14] De Franco E, Shaw-Smith C, Flanagan SE et al: GATA6 mutations cause a broad phenotypic spectrum of diabetes from pancreatic agenesis to adult-onset diabetes without exocrine insufficiency. Diabetes 2013; 62: 993–997. 23223019 10.2337/db12-0885 PMC3581234

